# DNA nanovaccines prepared using LemA antigen protect Golden Syrian hamsters against *Leptospira* lethal infection

**DOI:** 10.1590/0074-02760190396

**Published:** 2020-04-17

**Authors:** Thaís Larré Oliveira, Kátia Leston Bacelo, Karine Maciel Forster, Vinicius Ilha, Oscar Endrigo Rodrigues, Daiane D Hartwig

**Affiliations:** 1 Universidade Federal de Pelotas, Centro de Desenvolvimento Tecnológico, Programa de Pós-Graduação em Biotecnologia, Núcleo de Biotecnologia, Pelotas, RS, Brasil; 2 Universidade Federal de Santa Maria, Departamento de Química, Santa Maria, RS, Brasil; 3 Universidade Federal de Pelotas, Instituto de Biologia, Departamento de Microbiologia e Parasitologia, Pelotas, RS, Brasil

**Keywords:** Leptospira interrogans, nanoparticles, LIC11058, HNT, MWCNT

## Abstract

**BACKGROUND:**

Nanoparticles (NPs) are viable candidates as carriers of exogenous materials into cells via transfection and can be used in the DNA vaccination strategy against leptospirosis.

**OBJECTIVES:**

We evaluated the efficiency of halloysite clay nanotubes (HNTs) and amine-functionalised multi-walled carbon nanotubes (NH_2_-MWCNTs) in facilitating recombinant LemA antigen (rLemA) expression and protecting Golden Syrian hamsters (*Mesocricetus auratus*) against *Leptospira interrogans* lethal infection.

**METHODS:**

An indirect immunofluorescent technique was used to investigate the potency of HNTs and NH_2_-MWCNTs in enhancing the transfection and expression efficiency of the DNA vaccine in Chinese hamster ovary (CHO) cells. Hamsters were immunised with two doses of vaccines HNT-pTARGET/*lemA*, NH_2_-MWCNTs-pTARGET/*lemA*, pTARGET/*lemA*, and empty pTARGET (control), and the efficacy was determined in terms of humoral immune response and protection against a lethal challenge.

**FINDINGS:**

rLemA DNA vaccines carried by NPs were able to transfect CHO cells effectively, inducing IgG immune response in hamsters (p < 0.05), and did not exhibit cytotoxic effects. Furthermore, 83.3% of the hamsters immunised with NH_2_-MWCNTs-pTARGET/*lemA* were protected against the lethal challenge (p < 0.01), and 66.7% of hamsters immunised with HNT-pTARGET/*lemA* survived (p < 0.05).

**MAIN CONCLUSIONS:**

NH_2_-MWCNTs and HNTs can act as antigen carriers for mammalian cells and are suitable for DNA nanovaccine delivery.

Nanotechnology has elicited a high level of interest for various applications, including those of engineered nanovaccines. Nanoparticles (NPs) have a very important and promising application in vaccinology. New-generation vaccines have been proposed and evaluated against leptospirosis; these include subunit, genetic immunisation (DNA), and vectored vaccines.^1^ ^,^ ^2^ ^,^ ^3^

LemA is a putative lipoprotein that was identified using a reverse vaccinology approach.^4^ This protein presents an M3 epitope similar to *Listeria* and is conserved among the pathogenic *Leptospira* spp., and protects immunised hamsters (*Mesocricetus auratus*) when administered as a subunit (50.0-87.5%), DNA vaccine (62.5%), and prime-boost strategy (87.5%).^5^ ^,^ ^6^ These vaccines are safe with well-defined ingredients but contain less immunogenic antigens and hence require some adjuvants and suitable delivery systems to elicit robust immune responses. Low vaccine delivery rates may contribute to limited immunotherapy efficacy.

Several adjuvants/carriers have been evaluated for preparing leptospirosis vaccine formulations, *e.g.* Freund’s adjuvant,^7^ aluminum hydroxide,^5^ AddaVax^™^,^6^ CpG oligodeoxynucleotides (or CpG ODN), xanthan gum,^8^ mannosylated form,^9^ and NPs.^10^ ^,^ ^11^ NPs seemed to be potential antigen carriers for the development of new-generation vaccines, and combined nanovaccines have been known to induce more intense and long-lasting antigen-specific immune responses. NPs can induce a robust innate immunity response by causing abundant initial antigen exposure and long-term immunity through the sustained release of antigens via two types of activation modes: dendritic cell (DC) and T-cell differentiation.^12^ ^,^ ^13^ ^,^ ^14^ In previous studies, we demonstrated that subunit vaccines containing recombinant proteins, LipL32^10^ and LigAni,^11^ with halloysite nanotubes (HNTs) and carboxylated multi-walled carbon nanotubes (COOH-MWCNTs) as carriers induced a strong IgG immune response in Golden Syrian hamsters.

Several researchers designing drug delivery systems have considered biodegradable polymeric carriers to a great extent because of their low toxicity and side effects, and no accumulation in cells and tissues even after repeated administration.^15^ In this study, we evaluated HNTs and carbon nanotubes (CNTs) in the amine-functionalised multi-walled form (NH_2_-MWCNTs) as carriers of leptospirosis vaccine preparation. HNTs and MWCNTs have several advantages as vaccine nanocarriers; they can be produced at a large scale and at a low cost, can be easily functionalised, are biocompatible, can bear a large number of peptide ligands, and are immunostimulants.^16^ ^,^ ^17^ ^,^ ^18^

Over the last two decades, to increase the efficacy of vaccines against leptospirosis, several DNA vaccines have been formulated using novel strategies, such as the introduction of novel plasmid vectors, adjuvants, alternative delivery routes, and prime-boost regimens.^13^ DNA vaccines are promising because they can trigger both humoral and cellular immune responses, thus providing long-term protective immunity. However, naked DNA can hardly enter into cells and is easily degraded by DNases and lysosomes, thus highlighting the need for assessing effective delivery systems. DNA vaccines have been licensed to be used for veterinary medicine since 2005 in the USA. Furthermore, currently, there are a few veterinary DNA vaccines commercially available. However, in humans, clinical trials of DNA vaccines have yielded less than satisfactory results.^19^

Here, we describe for the first time DNA nanovaccines against leptospirosis in hamsters using HNTs and MWCNTs as delivery systems. We used these NPs as carriers for rLemA-based DNA vaccines and evaluated delivery *in vitro* and *in vivo* to determine their capacity to enhance the immune response against this antigen and protect hamsters against leptospirosis.

## MATERIALS AND METHODS

*Ethics statement* - All animal procedures were performed at the animal facility of the Federal University of Pelotas (UFPel) and approved by the Ethics Committee for Animal Experimentation (CEEA) of UFPel under protocol number 6255. The CEEA at UFPel is accredited by the Brazilian National Council for Animal Experimentation Control (CONCEA). The animals were maintained in accordance with international guidelines throughout the experiments.

*Materials* - HNTs and MWCNTs were obtained from Sigma^®^ (St. Louis, USA), and MWCNTs were functionalised at Departamento de Química, Universidade Federal de Santa Maria, Santa Maria, RS, Brazil.^20^ To increase their biocompatibility, we introduced amino groups on the surface of MWCNTs, as described previously.^21^ Characterisation of NH_2_-MWCNTs was performed using X-ray photoelectron spectroscopy (XPS) and Raman spectroscopy. Functionalised MWCNTs were found to be 60-70 nm in diameter and 1-2 µm in length.

*Nanotubes in vitro cytotoxicity* - The viability of Chinese hamster ovary (CHO) cells was determined by measuring the reduction of soluble 3-(4,5-dimethylthiazol-2-yl)-2,5-diphenyltetrazolium bromide (MTT) compared with water-insoluble formazan.^11^. Briefly, cells were seeded at a density of 2 × 10^4^ cells per well at a volume of 100 µL on 96-well plates and grown at 37ºC in a humidified atmosphere of 5% CO_2_ for 24 h prior to performing the cell viability assay. CHO cells were incubated with different concentrations of nanotubes (2.5-50.0 µg.mL^−1^) for 48 h. The media was removed and 180 µL of medium and 20 µL of MTT (5 mg MTT/mL solution) were added to each well. The plates were incubated for an additional 3 h and the medium was discarded. Dimethyl sulphoxide (200 µL) was added to each well, and formazan crystals were solubilised by shaking for 5 min at 100 × *g*. The absorbance of each well was read on a microplate reader (MR-96A, Mindray Shenzhen, China) at a wavelength of 492 nm. The cell inhibitory growth rate (%) was determined as follows: inhibitory rate = (1 - Abs_492treated cells_/Abs_492control cells_) × 100. All observations were validated by at least three independent experiments performed in triplicate.

*DNA vaccine preparation* - The pTARGET/*lemA* vector was constructed as described previously.^5^ *Escherichia coli* TOP10 competent cells were transformed with the pTARGET/*lemA* construct, and plasmid DNA was purified with the Perfectprep Plasmid Maxi kit (Eppendorf, Germany). Plasmid DNA concentration was determined with a Qubit Fluorometer (Invitrogen, Brazil).

*Nanotube transfection efficiency in mammalian cells* - To evaluate the transfection efficiency of HNTs and NH_2_-MWCNTs carrying the pTARGET/*lemA* vector, CHO cells were transfected with the constructs, HNT-pTARGET/*lemA*, NH_2_-MWCNTs-pTARGET/*lemA*, and pTARGET/*lemA*, using lipofectamine (Invitrogen) according to the manufacturer’s protocol. Briefly, CHO cells were maintained in flasks in Dulbecco’s modified Eagle’s medium (DMEM) supplemented with 100 IU/mL penicillin, 100 µg/mL streptomycin, and 10% (v/v) heat-inactivated foetal bovine serum (FBS). When a minimum of 50% confluence was reached, the cells were transfected with 2 µg of plasmid DNA in serum-free DMEM. Transfection with an empty control vector (pTARGET) as a negative control was performed. Forty-eight hours after transfection, the cells were collected, and their protein expression was analysed by indirect immunofluorescence. For this, the transfected cells were washed three times with phosphate buffer saline (PBS) at pH 7.4 and then fixed with methanol for 10 min at 4ºC. PBS plus 10% FBS was added and the slides were incubated for 30 min at 30ºC in a dark and humid chamber. Another washing with PBS plus FBS was performed and antibodies anti-LemA,^5^ diluted at a ratio of 1:100 in PBS were added, and the samples were incubated for 2 h at 4ºC in a dark and humid chamber. After washing with PBS plus FBS, 1:80 diluted anti-mouse FITC conjugate (Sigma) was added, and the cell suspension was incubated for 1 h at 30ºC. Then, another round of PBS plus FBS washing was performed, 10 µL of mounting medium was added, and the coverslips were sealed. The cells were visualised under fluorescence microscopy (Olympus) at an excitation wavelength of 450 nm.

*Immunisation of hamsters and challenge experiments* - Five-to-six-week-old female Golden Syrian Hamsters (*Mesocricetus auratus*) were divided into seven groups of six animals each. The vaccines used in this study were as follows: HNT-pTARGET/*lemA*, NH_2_-MWCNTs-pTARGET/*lemA*, pTARGET/*lemA*, HNT-pTARGET, NH_2_-MWCNTs-pTARGET, pTARGET, and a bacterin vaccine consisting of 1 × 10^9^ heat-killed whole-cells of *L. interrogans* serovar Copenhageni strain, Fiocruz L1-130. The vaccines were prepared 24 h before animal immunisations and preserved at 4ºC. The hamsters received a vaccine containing 50 µg of DNA mixed with 50 µg of carriers at day 0, and the booster dose was administered 21 days later. The hamsters were vaccinated by the intramuscular (IM) route in the hind leg. Blood samples were collected from the retro-orbital venous plexus before each immunisation and at 42 days post-immunisation (DPI), the sera were stored at -20ºC. Forty-two (42) days after the first immunisation, the hamsters’ immune systems were challenged with an intraperitoneal inoculum of 1.3 × 10^3^ leptospires of *L. interrogans* serovar Copenhageni strain, Fiocruz L1-130.^9^ Thereafter, the hamsters were monitored daily and euthanised when clinical signs of terminal disease appeared, such as prostration, ruffled fur, or weight loss of ≥ 10% of the animal’s maximum weight. The surviving hamsters were euthanised on day 30 post-challenge and the kidney tissue was used to inoculate the EMJH medium for culturing with the evaluation of sterilising immunity. Dark-field microscopy was performed during the eight-week incubation period to identify the positive cultures.

*Evaluation of the humoral immune response in hamsters* - Antibody responses were monitored by indirect enzyme-linked immunosorbent assay (ELISA) using rLemA. Each well was coated with 50 ng of rLemA diluted in carbonate-bicarbonate buffer, pH 9.6. The ELISA plates were washed three times with PBST [PBS with 0.05% (v/v) Tween 20] and then blocked. Hamster sera (diluted 1:50) was added for 1 h at 37ºC, and then the plates were washed three times with PBST. Anti-Golden Syrian Hamster IgG antibody peroxidase-conjugated (Rockland), at 1:8,000 dilution, was added, incubated at 37ºC for 1 h, washed five times with PBST, and the reaction was visualised with *o*-phenylenediamine dihydrochloride (Sigma-Aldrich) as well as hydrogen peroxide. The reaction was stopped by the addition of 0.1 M sulphuric acid, and absorbance was determined at 492 nm using a Multiskan MCC/340 ELISA plate reader (Titertek Instruments, USA).

*Statistical analysis* - Fisher’s exact test and the Wilcoxon log-rank test were used to determine significant differences for protection against mortality and survival, respectively. Significant differences in the serological, cytotoxic, and transfection assays were determined using analysis of variance (ANOVA). Differences were considered significant at a p-value of < 0.05. Statistical analysis was carried out using the Epi Info 6 (Centers for Disease Control, USA) and Prism 5 (GraphPad, USA) software packages.

## RESULTS

**Fig. 1:**
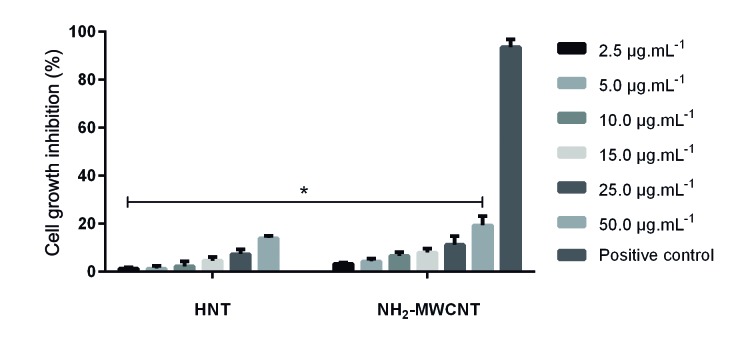
evaluation of cytotoxic effect of HNTs and NH_2_-MWCNTs in CHO cells by MTT assay. The inhibition rate was expressed as the optical density of treated cells compared to the negative control cells (only medium). The positive control cells were treated with 1% dimethyl sulphoxide. The data are expressed as mean ± SEM of three independent experiments. Asterisks indicate significant differences (p < 0.001) compared to the positive control.

*Nanotubes cytotoxicity* - The cytotoxicity of HNTs and NH_2_-MWCNTs was determined by the MTT assay (Fig. 1). The nanotubes did not affect cell growth even at a concentration of 50 µg.mL^−1^. All concentrations tested (2.5, 5.0, 10.0, 15.0, 25.0, and 50.0 µg.mL^−1^) presented inhibition rates statistically lower than that of the positive control treated with dimethyl sulphoxide (p < 0.001), not inhibiting more than 25% of the CHO cells.

**Fig. 2:**
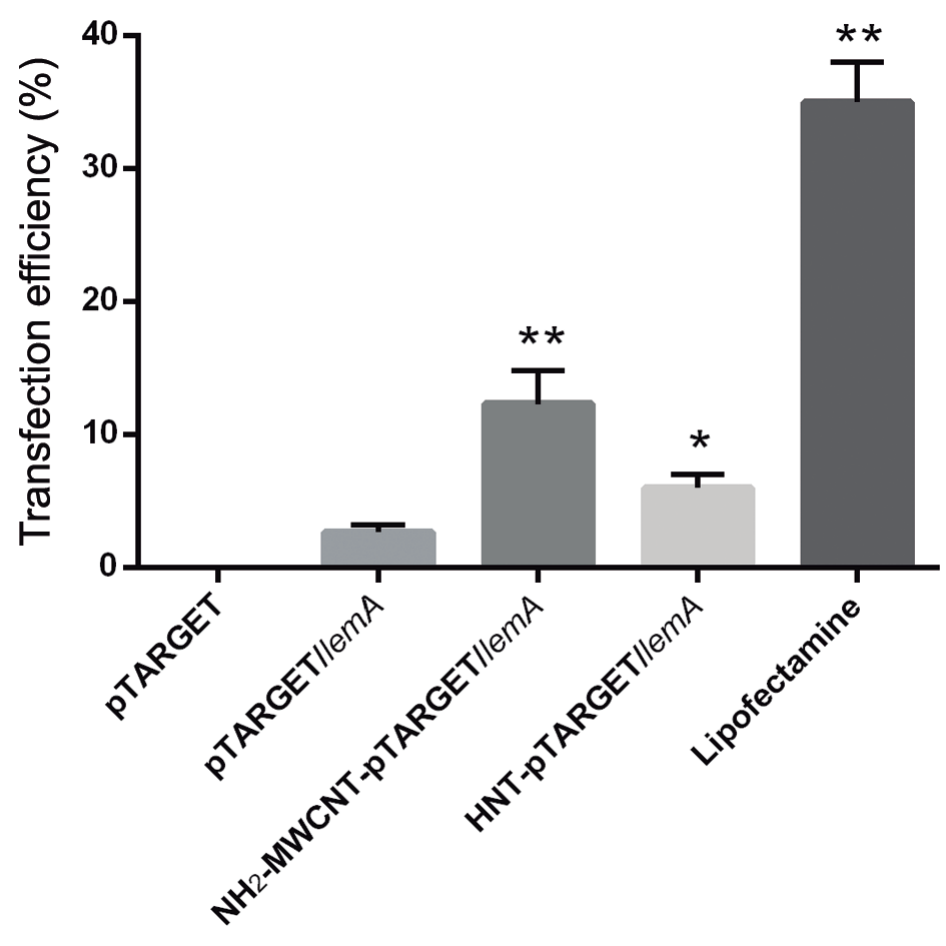
indirect immunofluorescent microscopy of CHO cells transfected with pTARGET, HNT-pTARGET/*lemA*, NH_2_-MWCNT-pTARGET/*lemA,* and pTARGET/*lemA* with and without lipofectamine (Invitrogen). The results were expressed as the percentage of cells with green fluorescence. Values are presented as means ± SEM of two independent experiments. Asterisks represent a difference between groups in comparison with pTARGET/*lemA* (*p < 0.05 and **p < 0.0001). The samples were analysed in triplicate.

*Transfection efficiency* - The ability of HNTs and NH_2_-MWCNTs in delivering the rLemA DNA vaccine to the CHO cells was evaluated by an indirect immunofluorescent technique using anti-rLemA antibodies (Fig. 2). The results were expressed as the percentage of the cells with green fluorescence, and the protein was successfully expressed in CHO cells. Compared to pTARGET/*lemA* alone, functionalised NH_2_-MWCNTs and lipofectamine (positive control) were more efficient in carrying the exogenous DNA into the cells (p < 0.0001), followed by HNT-pTARGET/*lemA* (p < 0.05). Fluorescence was not observed in control cells incubated with pTARGET (empty).

**Fig. 3:**
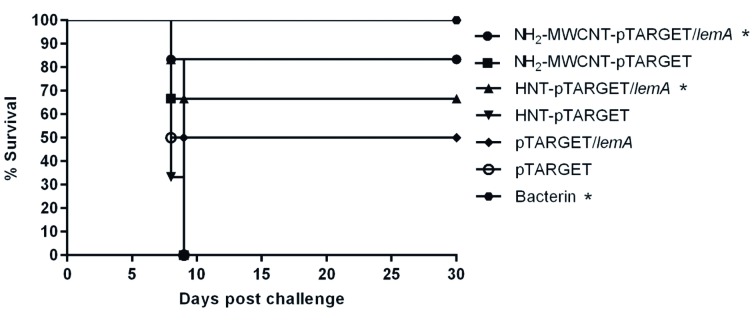
survival of hamsters immunised with rLemA-based DNA nanovaccines after challenge. Percent survival conferred by NH_2_-MWCNTs-pTARGET/*lemA*, HNT-pTARGET/*lemA,* and bacterin against lethal challenge was significant (p < 0.05*) in comparison to negative control groups. Survival curves were compared using the log rank (Mantel Cox test) analysis.

*Efficacy of DNA rLemA nanovaccines* - The protective efficacy of the nanovaccine preparations was determined by conducting challenge assays in hamsters. The results are presented in Fig. 3, which indicates that the nanovaccines were effective. The NH_2_-MWCNTs-pTARGET/*lemA* vaccine protected 83.3% of the hamsters against the lethal challenge (p < 0.05) and 66.7% of hamsters immunised with HNT-pTARGET/*lemA* survived (p < 0.05) (Fig. 3). Significance analyses were performed in comparison to the respective negative control group (NH_2_-MWCNTs-pTARGET and HNT-pTARGET). The hamsters immunised with the rLemA-based DNA vaccine (pTARGET/*lemA*) without nanocarriers were not significantly protected (50% of survival) against the challenge. All hamsters of the positive control group (bacterin) survived and all hamsters of the negative control groups (NH_2_-MWCNTs-pTARGET, HNT-pTARGET and pTARGET) died on days eight-nine after the challenge. All surviving animals immunised using the rLemA DNA vaccines were culture positive, indicating a lack of sterilising immunity.

**Fig. 4:**
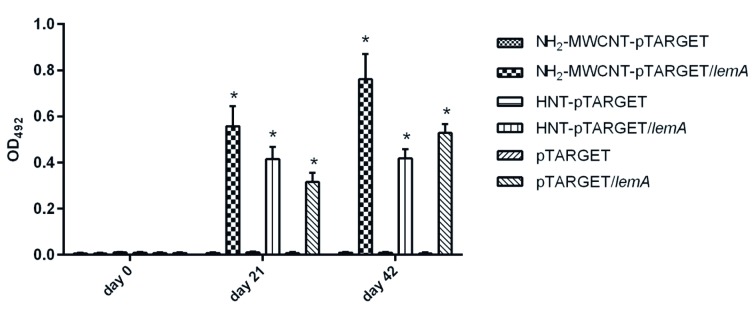
IgG antibody response in hamsters immunised with rLemA DNA nanovaccines. The specific IgG responses stimulated by the different immunogens were determined by an ELISA of the hamster serum diluted at 1:50, using rLemA produced in *Escherichia coli* as the antigen. Values are presented as means ± SEM of two independent experiments. Asterisk represent a difference compared to control groups (p < 0.05). The samples were analysed in triplicate.

*Evaluation of humoral immune response in hamsters* - The antibody profile induced by immunisation with the rLemA DNA nanovaccines was evaluated by ELISA using blood samples from hamsters immunised with either HNT-pTARGET/*lemA*, NH_2_-MWCNTs-pTARGET/*lemA*, pTARGET/*lemA*, HNT-pTARGET, NH_2_-MWCNTs-pTARGET, or pTARGET (Fig. 4). At 21 DPI, the IgG levels were enhanced for rLemA DNA vaccine preparations compared with those in the respective control groups (p < 0.05). At 42 DPI, the animals that received pTARGET/*lemA* administered with or without NPs showed significantly greater IgG response than the control groups (p < 0.05). Immunisations with HNTs-pTARGET, NH_2_-MWCNT-pTARGET, and pTARGET did not induce detectable levels of IgG antibodies.

## DISCUSSION

Vaccination is the most promising and effective strategy for infectious disease control. However, in the case of leptospirosis, the development of a safe, effective, and easy to formulate vaccine remains a challenge. Our group previously explored the use of new-generation vaccines in search of a successful vaccine for leptospirosis infection control. In this study, we report the use of a DNA vaccine prepared using LemA antigen and NP (NH_2_-MWCNTs and HNT) that increased transfection efficiency in mammalian cells and immunoprotection rates in hamsters exposed to *Leptospira* lethal infection.

LemA is a putative lipoprotein that was identified using a reverse vaccinology approach.^4^ The attachment of this protein to the extracellular matrix (laminin, fibrinogen, and collagen type IV) indicates that it plays a possible role in *Leptospira* adhesion during the infection process.^6^ LemA is present in different serovars and significantly protects hamsters when administered as subunit (50.0-87.5%), DNA (62.5%), or when vectored by rBCG under the control of different promoters (80-100%).^5^ ^,^ ^6^ ^,^ ^22^ Thus, the vaccine potential of this protein deserves to be better explored. In this study, vaccination with the rLemA-based DNA vaccine (pTARGET/*lemA*) protected 50% of the immunised hamsters, which was not significantly different from the results for the negative control (0% protection). When we used NPs as carriers, the protection rates increased significantly. The NH_2_-MWCNTs-pTARGET/*lemA* vaccine was able to protect 83.3% of the hamsters against the lethal challenge (p < 0.05), and 66.7% of hamsters immunised with HNT-pTARGET/*lemA* survived (p < 0.05).

The high protection rate using NPs for the delivery of rLemA-based DNA vaccines may be because of improved nanovaccine uptake and incorporation by mammalian cells. Many factors can affect NP uptake and incorporation such as the size of the NP, surface charge, hydrophobicity, and modifications on NPs.^23^ MWCNTs have the shape of a graphite sheet rolled into a cylinder and HNTs have a tube-like morphology, both exhibiting bioactive properties. The size of NPs plays a decisive role in the type of immune response induced. There are few reports on the effect of length of NPs with respect to biological activity, suggesting that bioactivity increases with length and diameter.^24^ ^,^ ^25^ ^,^ ^26^. Large NPs (> 500 nm) are mostly associated with dendritic cells from the injection site, and small NPs (< 200 nm) were found in dendritic cells and macrophages drained to the lymph node.^27^ The NH_2_-MWCNTs used in this study had a diameter of 60-70 nm and a length of 1-2 µm, while HNTs had a diameter of 30-70 nm and length of 1-3 μm and in association with the LemA DNA vaccine, were able to induce a significant IgG antibody responses in hamsters.

Several recombinant vaccines have been evaluated for leptospirosis control, but they were mostly composed of highly purified proteins (subunit vaccines) that mainly induce a humoral response. A mixed immune response involving both humoral and cellular stimulus is completer and more effective and is possible using genetic immunisation. In our study, the rLemA DNA vaccines using NPs as carriers were more effective in transfecting mammalian cells (CHO) and immunoprotecting hamsters against lethal infection when compared to the same vaccine without NPs. The IgG immune response induced by NP-rLemA protective vaccines was significantly higher than that induced by pTARGET/*lemA* alone. Furthermore, the antibody response against NH_2_-MWCNTs-pTARGET/*lemA* was more robust and different from that induced by HNT-pTARGET/*lemA*. In this study, we did not assess the involvement of cell-mediated immunity, and therefore, its involvement here is merely speculative.

Additionally, several studies have shown that functionalised CNTs do not cause cytotoxic effects^10^ ^,^ ^11^ ^,^ ^28^ ^,^ ^29^ as observed in this work. Amino-functionalised NPs were found to interact with negatively charged DNA and the cell membrane, and through this interaction, these successfully delivered the pTARGET/*lemA* plasmid into mammalian cells without any cytotoxicity. In a preliminary study, Gao and collaborators^16^ showed that DNA binding to NH_2_-MWCNTs owing to an electrostatic interaction between the positively changed NH_2_-MWCNT and negatively charged plasmid DNA. Thus, the functionalisation of NPs, such as the addition of amine groups, increases biocompatibility and mucoadhesive properties of these materials and, consequently, efficiency in delivering the antigen to host cells.^30^

To the best of our knowledge, this study is the first of its kind to demonstrate the potential of NPs as a delivery agent/adjuvant of a DNA vaccine against leptospirosis. The results presented here highlight the potential of NH_2_-MWCNTs and HNTs for the delivery of a rLemA DNA vaccine and protection of hamsters against pathogenic *Leptospira* infection and serve as evidence for a promising alternative with a safe, stable and easily manufactured vaccine.

## References

[1] Dellagostin OA, Grassmann AA, Hartwig DD, Felix SR, da Silva EF, McBride AJ (2011). Recombinant vaccines against leptospirosis. Hum Vaccin.

[2] Dellagostin OA, Grassmann AA, Rizzi C, Schuch RA, Jorge S, Oliveira TL (2017). Reverse vaccinology an approach for identifying leptospiral vaccine candidates. Int J Mol Sci.

[3] Grassmann AA, Kremer FS, dos Santos JC, Souza JD, Pinto LS, McBride AJA (2017). Discovery of novel leptospirosis vaccine candidates using reverse and structural vaccinology. Front Immunol.

[4] Hartwig DD, Seixas FK, Cerqueira GM, McBride AJ, Dellagostin AO (2011). Characterization of the immunogenic and antigenic potential of putative lipoproteins from Leptospira interrogans. Curr Microbiol.

[5] Hartwig DD, Forster KM, Oliveira TL, Amaral M, McBride AJ, Dellagostin OA (2013). A prime-boost strategy using the novel vaccine candidate, LemA, protects hamsters against leptospirosis. Clin Vaccine Immunol.

[6] Oliveira TL, Schuch RA, Inda GR, Roloff BC, Neto ACPS, Amaral M (2018). LemA and Erp Y-like recombinant proteins from Leptospira interrogans protect hamsters from challenge using AddaVax(tm) as adjuvant. Vaccine.

[7] Silva EF, Medeiros MA, McBride AJ, Matsunaga J, Esteves GS, Ramos JG (2007). The terminal portion of leptospiral immunoglobulin-like protein LigA confers protective immunity against lethal infection in the hamster model of leptospirosis. Vaccine.

[8] Bacelo KL, Hartwig DD, Seixas FK, Schuch R, Moreira AS, Amaral M (2014). Xanthan gum as an adjuvant in a subunit vaccine preparation against leptospirosis.. Biomed Res Int.

[9] Hartwig DD, Bacelo KL, Oliveira PD, Oliveira TL, Seixas FK, Amaral MG (2014). Mannosylated LigANI produced in Pichia pastoris protects hamsters against leptospirosis. Curr Microbiol.

[10] Hartwig DD, Bacelo KL, Oliveira TL, Schuch R, Seixas FK, Collares T (2015). The use of halloysite clay and carboxyl-functionalised multi-walled carbon nanotubes for recombinant LipL32 antigen delivery enhanced the IgG response. Mem Inst Oswaldo Cruz.

[11] Oliveira TL, Bacelo KL, Schuch R, Seixas FK, Collares T, Rodrigues OED (2016). Immune response in hamsters immunised with a recombinant fragment of LigA from Leptospira interrogans, associated with carrier molecules. Mem Inst Oswaldo Cruz.

[12] Rahimian S, Kleinovink JW, Fransen MF, Mezzanotte L, Gold H, Wisse P (2015). Near-infrared labeled, ovalbumin loaded polymeric nanoparticles based on a hydrophilic polyester as model vaccine in vivo tracking and evaluation of antigen-specific CD8(+) T cell immune response. Biomaterials.

[13] Silveira MM, Oliveira TL, Schuch RA, McBride AJA, Dellagostin OA, Hartwig DD (2017). DNA vaccines against leptospirosis a literature review. Vaccine.

[14] Noh YW, Jang YS, Ahn KJ, Lim YT, Chung BH (2011). Simultaneous in vivo tracking of dendritic cells and priming of an antigen-specific immune response. Biomaterials.

[15] Kalam MA, Khan AA, Alshamsan A (2017). Non-invasive administration of biodegradable nano-carrier vaccines. Am J Transl Res.

[16] Gao L, Nie L, Wang T, Qin Y, Guo Z, D Yang (2006). Carbon nanotube delivery of the GFP gene into mammalian cells. Chembiochem.

[17] Pantarotto D, Partidos CD, Hoebeke J, Brown F, Kramer E, Briand JP (2003). Immunization with peptide-functionalized carbon nanotubes enhances virus-specific neutralizing antibody responses. Chem Biol.

[18] Vergaro V, Abdullayev E, Lvov YM, Zeitoun A, Cingolani R, Rinaldi R (2010). Cytocompatibility and uptake of halloysite clay nanotubes. Biomacromolecules.

[19] Porter KR, Raviprakash K (2017). DNA vaccine delivery and improved immunogenicity. Curr Issues Mol Biol.

[20] Stéfani D, Paula AJ, Vaz BG, Silva RA, Andrade NF, Justo GZ (2011). Structural and proactive safety aspects of oxidation debris from multiwalled carbon nanotubes. J Hazard Mater.

[21] Chidawanyika W, Nyokong T (2010). Characterization of amine-functionalized single-walled carbon nanotube-low symmetry phthalocyanine conjugates. Carbon.

[22] Oliveira TL, Rizzi C, da Cunha CEP, Dorneles J, Seixas ACP, Amaral MG (2019). Recombinant BCG strains expressing chimeric proteins derived from Leptospira protect hamsters against leptospirosis. Vaccine.

[23] Hayat SMG, Darroudi M (2019). Nanovaccine a novel approach in immunization. J Cell Physiol.

[24] Donaldson K, Murphy FA, Duffin R, Poland CA (2010). Asbestos, carbon nanotubes and the pleural mesothelium a review of the hypothesis regarding the role of long fibre retention in the parietal pleura, inflammation and mesothelioma. Part Fibre Toxicol.

[25] Palomäki J, Välimäki E, Sund J, Vippola M, Clausen PA, Jensen KA (2011). Long, needle-like carbon nanotubes and asbestos activate the NLRP3 inflammasome through a similar mechanism. ACS Nano.

[26] Bussy C, Paineau E, Cambedouzou J, Brun N, Mory C, Fayard B (2012). Critical role of surface chemical modifications induced by length shortening on multi-walled carbon nanotubes-induced toxicity. Part Fibre Toxicol.

[27] Manolova V, Flace A, Bauer M, Schwarz K, Saudan P, Bachmann MF (2008). Nanoparticles target distinct dendritic cell populations according to their size.. Eur J Immunol.

[28] Pantarotto D, Partidos CD, Graff R, Hoebeke J, Briand JP, Prato M (2003). Synthesis, structural characterization, and immunological properties of carbon nanotubes functionalized with peptides. J Am Chem Soc.

[29] Zeinali M, Jammalan M, Ardestani SK, Mosaveri N (2009). Immunological and cytotoxicological characterization of tuberculin purified protein derivative (PPD) conjugated to single-walled carbon nanotubes. Immunol Lett.

[30] Rieger C, Kunhardt D, Kaufmann A, Schendel D, Huebner D, Erdmann K (2015). Characterization of different carbon nanotubes for the development of a mucoadhesive drug delivery system for intravesical treatment of bladder cancer. Int J Pharm.

